# Structure-Antifouling Activity Relationship and Molecular Targets of Bio-Inspired(thio)xanthones

**DOI:** 10.3390/biom10081126

**Published:** 2020-07-30

**Authors:** Joana R. Almeida, Andreia Palmeira, Alexandre Campos, Isabel Cunha, Micaela Freitas, Aldo Barreiro Felpeto, Maria V. Turkina, Vitor Vasconcelos, Madalena Pinto, Marta Correia-da-Silva, Emília Sousa

**Affiliations:** 1 CIIMAR/CIMAR—Interdisciplinary Centre of Marine and Environmental Research, University of Porto, Avenida General Norton de Matos, 4450-208 Matosinhos, Portugal; joana.reis.almeida@gmail.com (J.R.A.); andreiapalmeira@gmail.com (A.P.); amoclclix@gmail.com (A.C.); isabel.cunha@ciimar.up.pt (I.C.); Micaela.Faria@unige.ch (M.F.); aldo.barreiro@gmail.com (A.B.F.); vmvascon@fc.up.pt (V.V.); madalena@ff.up.pt (M.P.); esousa@ff.up.pt (E.S.); 2 Laboratory of Organic and Pharmaceutical Chemistry, Department of Chemical Sciences, Faculty of Pharmacy, University of Porto, Rua Jorge Viterbo Ferreira, 228, 4050-313 Porto, Portugal; 3 Department of Biology, Faculty of Sciences, University of Porto, Rua do Campo Alegre, P 4069-007 Porto, Portugal; 4 ISPSO—Institut des Sciences Pharmaceutiques de Suisse Occidentale, University of Geneva, 1205 Geneva, Switzerland; 5 Department of Biomedical and Clinical Sciences, Linköping University, SE-581 85 Linköping, Sweden; maria.turkina@liu.se

**Keywords:** xanthones, chemical synthesis, antifouling activity, invertebrates, molecular targets

## Abstract

The development of alternative ecological and effective antifouling technologies is still challenging. Synthesis of nature-inspired compounds has been exploited, given the potential to assure commercial supplies of potential ecofriendly antifouling agents. In this direction, the antifouling activity of a series of nineteen synthetic small molecules, with chemical similarities with natural products, were exploited in this work. Six (**4**, **5**, **7**, **10**, **15** and **17**) of the tested xanthones showed in vivo activity toward the settlement of *Mytilus galloprovincialis* larvae (EC_50_: 3.53–28.60 µM) and low toxicity to this macrofouling species (LC_50_ > 500 µM and LC_50_/EC_50_: 17.42–141.64), and two of them (**7** and **10**) showed no general marine ecotoxicity (<10% of *Artemia salina* mortality) after 48 h of exposure. Regarding the mechanism of action in mussel larvae, the best performance compounds **4** and **5** might be acting by the inhibition of acetylcholinesterase activity (in vitro and in silico studies), while **7** and **10** showed specific targets (proteomic studies) directly related with the mussel adhesive structure (byssal threads), given by the alterations in the expression of *Mytilus* collagen proteins (PreCols) and proximal thread proteins (TMPs). A quantitative structure-activity relationship (QSAR) model was built with predictive capacity to enable speeding the design of new potential active compounds.

## 1. Introduction

Biofouling is the temporary or permanent adhesion of organisms on water submerged man-made surfaces. It starts with the adsorption of a film of molecules and particles a few minutes after immersion, followed by the adhesion of bacteria, cyanobacteria, unicellular algae, and protozoa that form biofilms (microfouling) [1]. These organisms release biochemical cues that transmit specific information to the environment [2]. Such cues reach conspecifics and also organisms of other species, influencing the settlement, and metamorphosis of specific macrofouling species [3].

Maritime industries make large investments worldwide to control biofouling, which constitute an economic burden for shipping and aquaculture [4], and also to non-maritime industries such as paper manufacturing, food processing, underwater construction, power plants, and others. On the other hand, environmental problems are also associated with biofouling as the introduction of invasive species via vessels and maritime platforms promotes unbalances on local marine communities and contributes to loss of marine biodiversity [5].

The most common strategy being applied to combat biofouling is the use of antifouling coatings based on effective but somehow toxic active principles that need to be replaced [6,7], and environmentally benign alternatives are needed.

Natural alternatives including primary or secondary metabolites from a multitude of species have been found to inhibit the settlement of different biofouling species [8,9,10,11], particularly polyketide-related compounds [9]. Xanthone polyketide-derived compounds represent a class of marine natural compounds with interesting biological effects. Their interesting structural scaffold and the significant biological activities has led to the description of xanthones as “privileged structures” [12]. Several members were found to have antimicrobial activity and weak toxicity to brine shrimp (*Artemia salina*) [13]. Two new xanthone derivatives isolated from a coral-derived *Aspergillus sp*. were identified as having antifouling activities against *Balanus amphitrite* (EC_50_ < 0.39 μM) [14]. More recently, a xanthone isolated from a marine-derived fungus *Aspergillus terreus* was also found to be a potent antifoulant [15]. Nonetheless, little information on structure–antifouling activity relationship and ultimately on the mechanism of action of polyketide-related compounds, namely xanthones, is available [16,17].

**Figure 1:**
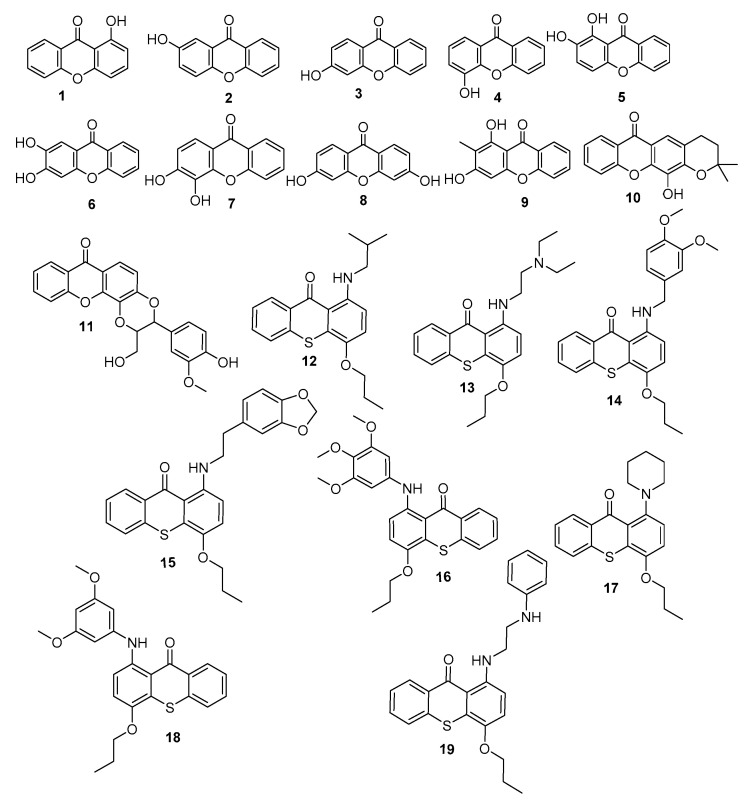
Library of xanthones investigated for antifouling activity.

Based on the above, and as a part of our efforts to discover innovative antifoulants inspired in natural products [18,19,20], a library of 19 synthetic xanthones was investigated for their antifouling potential (Figure 1).

The anti-settlement activity toward the macrofouling species *Mytilus galloprovincialis* was evaluated in vivo and the subjacent mechanism of action of the most promising compounds was pursued by the potential modulation of selected enzymes with a role in adhesive processes (acetylcholinesterases (AChE) and tyrosinase (Tyr)) and by the differential analysis of the proteome of *M. galloprovoincialis* plantigrade larvae after exposure. In silico studies were also performed to disclose a quantitative structure–activity relationship (QSAR). *Artemia* ecotoxicity bioassay was performed to assess general marine ecotoxicity of antifouling candidates.

## 2. Materials and Methods

### 2.1. Chemical Syntheses

Melting points were obtained in a Köfler microscope (Wagner and Munz, Munich, Germany) and are uncorrected. IR spectra were recorded on a Nicolet iS10 FTIR spectrometer (ThermoScientific, ThermalSciecntific, MA, USA) in KBr microplates. ^1^H NMR spectra were taken in DMSO-d_6_ at room temperature, on Bruker DRX 300 instrument. Chemical shifts are expressed in δ (ppm) values relative to TMS. The following 19 xanthone derivatives were synthesized according to previously described procedures [21,22,23,24]: 1-hydroxy- (**1**), 2-hydroxy-(**2**), 3-hydroxy-(**3**), 4-hydroxy-(**4**), 1,2-dihydroxy-(**5**), 2,3-dihydroxy-(**6**), 3,4-dihydroxy-(**7**), 3,6-dihydroxy-(**8**), 1,3-dihydroxy-2-methyl-(**9**), 4-dihydro-12-hydroxy-2,2-dimethyl-2*H*,6*H*-pyrano [3,2-b]xanthone (**10**), 2,3-dihydro-3-(4-hydroxy-3-methoxyphenyl)-2-(hydroxymethyl)-7*H*-1,4-dioxino[2,3-c]- (**11**). The synthesis of the following thioxanthone derivatives was performed according to described procedures [25]: 1-(Isobutylamino)-4-propoxy-9*H*-thioxanthen-9-one (**12**), 1-((2-(diethylamino)ethyl)amino)-4-propoxy-9*H*-thioxanthen-9-one (**13**), 1-[(3,4-dimethoxybenzyl)amino]- 4-propoxy-9*H*-thioxanthen-9-one (**14**), 1-[11]-4-propoxy-9*H*-thioxanthen-9-one (**15**), 1-[(3,4,5-trimethoxyphenyl)amino]-4-propoxy-9*H*-thioxanthen-9-one (**16**), 1-(piperidin-1-yl)-4-propoxy-9*H*-thioxanthen-9-one (**17**), 1-[(3,5-dimethoxyphenyl)amino]-4- propoxy-9H-thioxanthen-9-one (**18**), and 1-[2-(phenylamino)ethyl]amino]-4-propoxy-9*H*-thioxanthen-9-one (**19**). The investigated compounds presented purity > 95% by HPLC-DAD.

### 2.2. Antifouling Screening

#### 2.2.1. *M. galloprovincialis* Larvae Anti-Settlement Bioassays

The 19 xanthone derivatives were screened for anti-settlement activity toward *M. galloprovincialis* plantigrade larvae. Samples of *M. galloprovincialis* juveniles were collected from mussel beds during low tides in the intertidal zone of Memória beach, Matosinhos, Portugal (41°13′59″ N; 8°43′28″ W). Mussel plantigrade larvae (0.5–2 mm) were sorted in a binocular magnifier (Olympus SZX2-ILLT, Tokyo, Japan) and kept in filtered seawater until bioassays preparation. Mussel plantigrades exhibiting foot exploring behavior were selected for the xanthone derivatives screening bioassay at 50 µM performed in 24-well microplates for 15 h, in the dark at 18 ± 1 °C, following Almeida et al. (2015) [26]. Five competent larvae were included per well and four well replicates per condition. Stock solutions (50 mM) in DMSO were used to prepare test media in filtered seawater (0.1%). Negative and positive controls were used, namely DMSO (0.1%) and CuSO_4_ (5 μM) as an efficient antifouling agent. After exposure, the anti-settlement bioactivity was determined by the observation of efficiently attached plantigrade larvae considering the existence/absence of produced byssal threads for each test condition. Compounds producing more than 50% of settlement inhibition at 50 µM were selected for antifouling effectiveness (EC_50_) and toxicity (LC_50_ and therapeutic index) bioassays, according to previously validated experimental conditions.

#### 2.2.2. Quantitative Structure-Activity Relationship Model

Nineteen (thio)xanthones were used to construct a QSAR model using the biological data obtained from the in vitro antifouling activity studies, measured as log (100/%attachment). Antifouling activity was selected as dependent variable in the QSAR analysis. The 19 molecules were randomly distributed into a training set (15 molecules) and a test set (4 molecules). CODESSA 2.7.10 (CompuDrug, University of Florida, Gainesville, FL, USA) was used to calculate hundreds of constitutional, topological, geometrical, electrostatic, quantum-chemical, and thermodynamical molecular descriptors. The heuristic multilinear regression protocol was used to execute a thorough quest for the best multilinear correlations with the descriptors of the training set. The 2D-QSAR model with the best correlation coefficient (R^2^), F-test (F), and standard error (s) was chosen. The final model was farther validated using the test set and the leave-one-out cross validation.

### 2.3. Antifouling Mechanism of Action

#### 2.3.1. In Vitro Determination of Acetylcholinesterase (AChE) Inhibition Activity

AChE inhibition was evaluated as a neurotransmission biomarker for the 19 compounds using *Electrophorus electric* AChE Type V-S (SIGMA C2888, E.C. 3.1.1.7), according to Ellman et al. (1961) [27] and modified by Almeida et al. (2015) [26]. Briefly, 250 μL of the reaction solution containing 30 mL of phosphate buffer, 1mL of the reagent dithiobisnitrobenzoate (DTNB) at 10 mM (acid dithiobisnitrobenzoate and sodium hydrogen carbonate in phosphate buffer), and 200 μL of acetylcholine iodide 0.075 M was added to 50 μL of pure AChE (0.25 U.mL^−1^) and each test compound at final concentration of 30 μg mL^−1^ in quadruplicate. AChE activity was followed at 412 nm during 5 min at 25 °C. Negative and positive controls were included, namely ultra-pure water and eserine (20 mM), respectively, and a blank containing 50 μL of phosphate buffer.

#### 2.3.2. In Vitro Determination of Tyrosinase Inhibition Activity

Tyrosinase inhibition assays were conducted using *Agaricus bisporus* tyrosinase (EC 1.14.18.1) according to Adhikari et al. [28] with appropriate adaptations. The enzymatic reaction follows the catalytic conversion of l-Dopa to dopaquinone and the formation of dopachrome by measuring the absorbance at 475 nm. Briefly, 50 µL of tyrosinase (25 U.mL^−1^) in 50 mM phosphate buffer pH 6.5, adding the defined concentration of compounds in DMSO. The enzymatic activity was initiated by l-dopa (25 mM). Kojic acid was used as positive control and DMSO as negative control.

#### 2.3.3. Docking Studies for AChE Activity

Docking studies were conducted by modulation of *Electrophorus electric* AChE and *Agaricus bisporus* tyrosinase. Crystal structure of *Electrophorus electric* AChE (PDB code: 1C2O) and of *Agaricus bisporus* tyrosinase (PDB code: 2Y9X), downloaded from the protein databank (PDB), were used for the docking studies. Structure files of two known AChE inhibitors described as antifouling agents, pulmonarins A and B [29], and seven known tyrosinase inhibitors also described as antifouling agents, PLOS12-18 [30], were drawn and minimized using the chemical structure drawing tool Hyperchem 7.5 (Hypercube). Autodock Vina (Molecular Graphics Lab, The Scripps Research Institute, San Diego, CA, USA). was employed for the docking study, using the following parameters: exhaustiveness = 8; grid box = 25.0 × 25.0 × 25.0 Å, surrounding the acetylcholine active site and peripheral anionic site (PAS); grid box = 15.0 × 20.0 × 25.0 Å, surrounding the crystallographic ligand binding site of tyrosinase. Top 9 poses were found for each input molecule. The lowest docking score poses that fit the active site triad (Ser-203, His-447, and Glu-334) and PAS of AChE, and active site of tyrosinase containing two copper atoms coordinated with histidines were chosen for further analysis of binding mechanism and visual inspection using PyMol 1.3 (Schrödinger, New York, NY, USA).

#### 2.3.4. Antifouling Molecular Targets Assessment in *M. galloprovincialis* by Differential Proteome Analysis

*M. galloprovincialis* plantigrade larvae proteome was analyzed according to experimental procedures described in Campos et al. (2016) [31]. Briefly, the whole protein content of *M. galloprovincialis* plantigrades (10 larvae per replicate) was solubilized in lysis buffer with 2% (*w*/*v*) SDS, 100 mM Tris-HCl, 0.1 M DTT, and protease inhibitor at pH 7.6. Proteins were subsequently digested using the filter aided sample preparation (FASP) [32]. The obtained peptides were analyzed by nano-LC coupled to a hybrid Ion trap-Orbitrap mass spectrometer (LTQ Orbitrap Velos Pro, Thermo Scientific, Waltham, EUA). Full scans were performed at 30,000 resolution with scan ranges of 380–2000 *m*/*z* and the top 20 most intense ions isolated and fragmented. Collision-induced fragmentation (CID) was used to fragment precursor ions. LTQ raw data were first processed in Proteome Discoverer software (version 1.4, Thermo Scientific, Waltham, EUA) and identifications achieved using X!Tandem algorithm in the Scaffold program (version Scaffold 4.3.4, Proteome Software, Portland, OR, USA) and using a composite database built with genomic and transcriptomic information from *M. gallopronvicialis* (46,791 sequences) and *Bathymodiolus azoricus* (33,464 sequences) [31]. Protein quantitative analysis was determined using normalized spectral abundance factors (NSAFs) in Scaffold program and employing non-parametric statistics (*p* < 0.05).

### 2.4. Ecotoxicity Assessment

Ecotoxicity to non-target species was assessed using the brine shrimp (*Artemia salina*) lethality test standard protocol [18,33]. Artemia cysts were cultivated and newly hatched nauplii I were used for the exposure to the most promising antifouling compounds at 50 and 25 µM. The bioassay was performed in 96-well microplates with eight replicates per condition and 15–20 nauplii per well. Positive (K_2_Cr_2_O_7_ at 13.6 µM) and negative (DMSO) controls were included. After 48 h of exposure, the percentage of nauplii mortality was determined.

### 2.5. Data Analysis

Data from the anti-settlement bioassays, namely the semi-maximum response concentration that inhibited 50% of larval settlement (EC_50_) for each bioactive compound was assessed using Probit regression analysis (Log10) with 95% lower and upper confidence limits [95% LCL;UCL]. Therapeutic ratios (LC_50_/EC_50_) were used to evaluate the effectiveness versus toxicity of bioactive compounds. For the differential proteome analysis, non-parametric Kruskall-Wallis (KW) and Mann Whitney (MW) tests were utilized to report protein abundance differences between treatments in MultiExperiment Viewer (MeV) version 4.9 (The Institute for Genomic Research (TIGR), La Jolla, CA, USA) at a confidence level of 95% [34]. The hierarchical clustering of proteins was made using Pearson correlation and average linkage method.

## 3. Results and Discussion

### 3.1. Antifouling Bioactivity 

From the series of compounds tested (**1–19**), which include 11 xanthones (oxygenated, prenylated, lignoids) and 8 aminated thioxanthones, significant anti-settlement responses (≥50% of settlement inhibition) were identified in the initial screening bioassay in the presence of six compounds, namely **4** (70.0 ± 12.9%); **5** (70.0 ± 12.9%); **7** (65.0 ± 17.1%); **10** (75 ± 9.6%); **15** (71.2 ± 10.9%); and **17** (71.7 ± 5.0%). These compounds were selected for dose-response studies.

**Figure 2:**
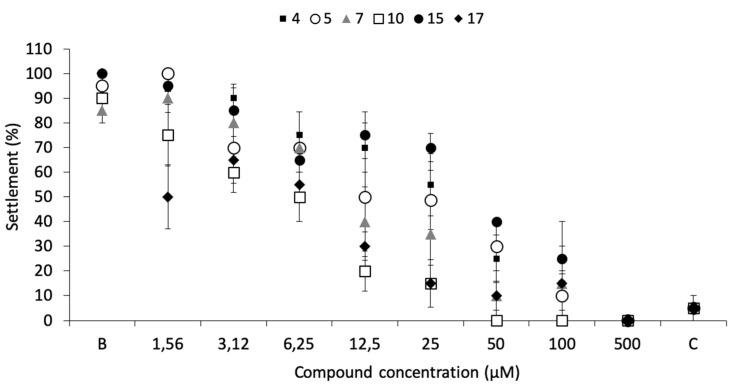
Dose-response of anti-settlement activity of the promising antifouling compounds **4**, **5**, **7**, **10**, **15**, and **17** toward plantigrades of the mussel *M. galloprovincialis.* B: negative control; C: CuSO_4_ 5 µM as positive control.

Dose-response bioassays confirmed the antifouling bioactivity toward mussel plantigrade larvae settlement, being the most effective the compounds **17** (EC_50_ = 3.53 µM), **10** (EC_50_ = 4.60 µM), and **7** (EC_50_ = 11.53 µM) (Figure 2; Table 1).

Regarding toxicity to mussel plantigrade larvae, only compounds **10** and **17** showed some mortality at high concentrations, 15% at 100 µM and 17% at 500 µM, respectively, a much lower mortality when compared to the commercial eco-friendly active ingredient ECONEA (50% at 108 µM), in the same bioassay conditions [20].

No mortality was observed for all the other promising compounds at any of the concentrations tested. Therapeutic ratios (LC_50_/EC_50_) were higher than 15 for all the compounds, the most promising levels being related to compounds **7**, **10,** and **17** (Table 1). These results highlighted the importance of a hydroxyl substituent at position 4 of the xanthonic scaffold.

### 3.2. Structure-Activity Relationship (QSAR) Studies

As quantitative structure-activity relationship (QSAR) studies have been employed during decades to find relevant small molecules properties and to forecast different biological activities [35], a QSAR model was built to highlight the descriptors important for the anti-macrofouling activity of these derivatives. In this work, a 2D-QSAR model was built using Comprehensive Descriptors for Structural and Statistical Analysis (CODESSA) software package (CompuDrug, CODESSA software version 2.7.2, University of Florida, Gainesville, FL, USA). A huge number of constitutional, topological, geometrical, electrostatic, and quantum-chemical descriptors are automatically generated. The heuristic method continues with a preselection of descriptors by erasing those that are not available for each structure, that have a small variation in magnitude for all structures, that are correlated pairwise, and that have no statistical significance. The heuristic method is a quick and very appropriate method for searching the best group of descriptors.

**Figure 3:**
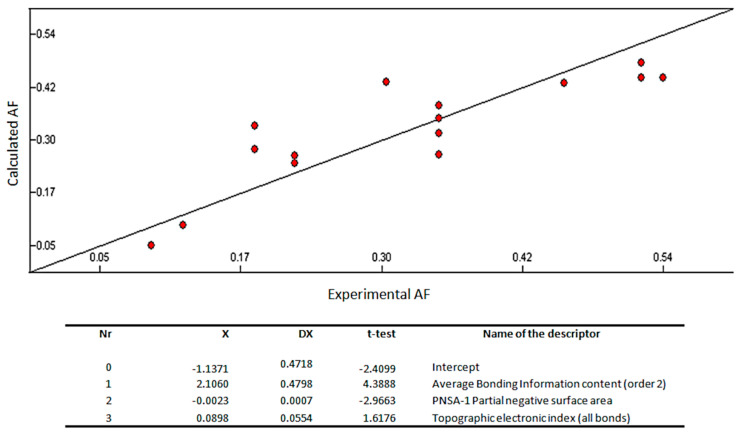
Quantitative structure–activity relationship (QSAR) model obtained with the heuristic method for 15 xanthones with the CODESSA software (R^2^ = 0.7335, F = 10.09, and s = 0.0085). X, ΔX and t-test are the regression coefficient of the linear model, standard errors of the regression coefficient, and the t significance coefficient of the determination, respectively. AF = antifouling activity.

The correlation coefficient (R^2^), standard error (s), and Fisher’s value (F) were used to evaluate the validity of regression equation. As the training set was composed of 15 molecules, 3 descriptors were used to build the QSAR model. The multilinear regression analysis using the Heuristic method for 15 compounds in the three-parameter model is given in Figure 3.

The best training model had a quality (R^2^) of 0.7335, Fisher value of 10.09, and S of 0.0085, which is an evidence that the suggested model has suitable statistical stability and validity. The R^2^, a relative measure of quality of the model, is greater than 0.7, which is a signal of an appropriate fit to the regression line. Accordingly, it represents more than 70% of the total variance in antifouling activity shown by the test compounds. The F-test represents the ratio of the variance of the model and the variance due to the error in the regression. The QSAR model is statistically significant at the 95% level as revealed by the F-test values which are higher than the tabulated values (3.59). Standard deviation s is a measurement of the quality of the fit and should have a low value (0.0085) for the regression to be significant. The leave-one-out cross-validated R^2^ (Q^2^) assesses how the results of the QSAR model will generalize to an independent data set. Q^2^ (0.57) is smaller than the overall R^2^ (0.73), but still the difference between R^2^ and Q^2^ is lower than 0.3, which indicates that the model has suitable predictivity. External test set predictivity was also used to validate the model. The model was capable of predicting the growth inhibitory activity of an external test set with an average difference of 0.15 from the experimental results [36]. From everything before mentioned, it can be said that the QSAR model is relevant for the prediction of the antifouling activity of other compounds.

Average bonding information content (order 2), PNSA-1 partial negative surface area, and topographic electronic index (all bonds) are predicted as being involved in the antifouling activity of the tested compound (Figure 3).

A graph vertex complexity index descriptor—average bonding information content (order 2)—is predicted as being the most relevant descriptor affecting positively the antifouling activity of the test compounds. It is a symmetry descriptor (neighborhood symmetry of second order) related to the number of bonds counting bond orders, which provides information on the complexity of the molecule [37,38,39].

The second most important descriptor is the partial negative surface area PNSA-1, which quantifies the actually available surface area portion provided by all negatively charged atoms [39].

The third most important descriptor is an electrostatic-related descriptor—topographic electronic index—that is calculated from the partial atomic charges and the inter-atomic distances between all the pairs of atoms in the molecule. This type of descriptor reflects how differences in size, shape, and constitution affect the electronic charge distribution and inter-atomic distances of the molecules [39].

In summary, the structure-activity relationship captured by the linear model indicates that the complexity of the molecule, the electronic charge distribution, and inter-atomic distances influence the antifouling activity.

### 3.3. Insights on Antifouling Mode of Action

#### 3.3.1. AChE Activity

**Figure 4:**
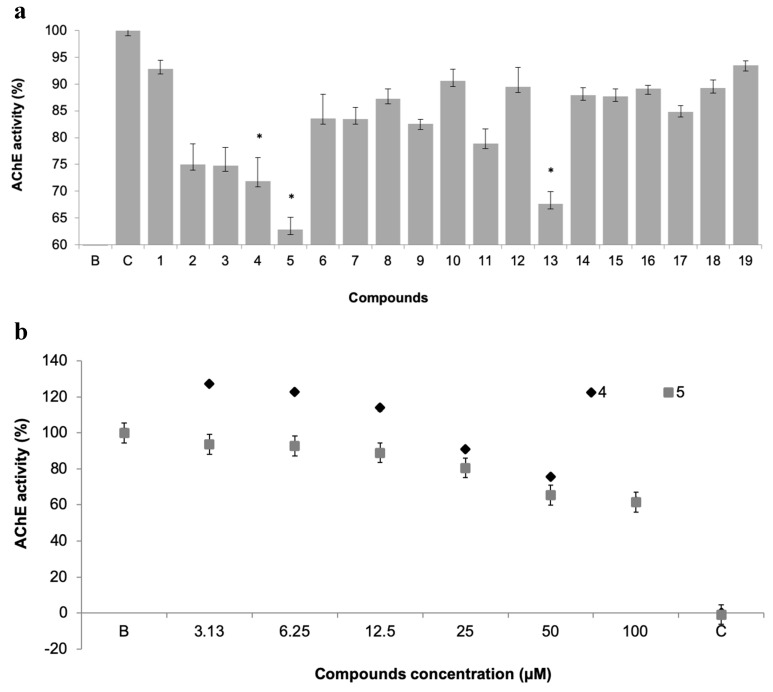
(**a**) Acetylcholinesterases (AChE) activity of 19 pure xanthones (50 µM). * significant differences (*p* ≤ 0.05, Dunnett’s test) compared to the negative control (B); eserine was used as positive control (C). (**b**) Dose-response of AChE activity of selected xanthones **4** and **5**.

The potential of xanthone derivatives to inhibit AChE activity was evaluated for the 19 xanthones, as AChE has a metabolic role in the process of settlement of macrofouling organisms (Figure 4a) [26].

Three compounds showed AChE inhibition at 50 µM when compared to control, namely the promising antifouling xanthones **4** and **5**, which showed concentration-dependent modulation of AChE activity (Figure 4b). This inhibitory activity may be contributing to the antifouling activity of these two compounds.

#### 3.3.2. Docking Studies Regarding AChE Activity

**Figure 5:**
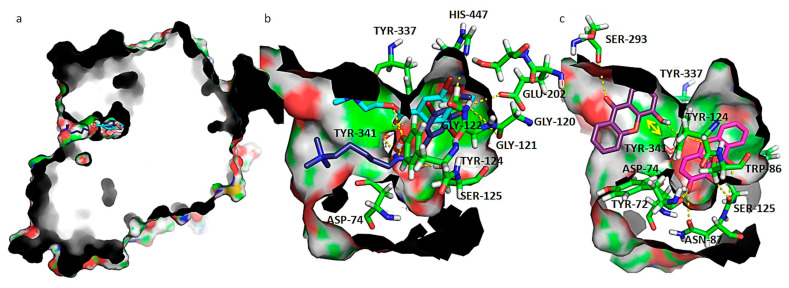
(**a**) Capped surface of AChE, showing the ACh binding pocket with AChE inhibitors pulmonarin A and B (blue sticks); (**b**) detailed view of known antifouling AChE inhibitors: pulmonarin A (light blue sticks) and pulmonarin B (dark blue sticks); (**c**) detailed view of test compounds **4** (purple sticks) and **5** (magenta sticks). Hydrogen interactions are represented with yellow broken line and stacking interactions with a double edge yellow arrow. Residues involved on those interactions are represented as green sticks and labeled. AChE is represented as solid surface, where carbon, hydrogen, oxygen, nitrogen, and sulfur are represented in green, grey, red, blue, and yellow, respectively.

In order to understand the binding mode of the most promising molecules **4** and **5** to AChE, docking studies were performed. AChE is a serine hydrolase and the crystallographic structure reveals the presence of a narrow, long, and hydrophobic cavity (Figure 5). The active site of AChE comprises 2 subsites—the esteratic and anionic subsites. The esteratic subsite, where acetylcholine (ACh) is hydrolyzed to acetate and choline, contains the catalytic triad of three amino acids: Ser-203, His-447, and Glu-334, the lining of which contains mostly aromatic residues that form a narrow entrance to the catalytic Ser-203 [40]. The activation of Ser-203 allows the acylation between hydroxyl group of that residue and ACh oxygen. In the anionic site, the indole side chain of the conserved residue Trp86 makes a cation-π interaction with the quaternary amino group of ACh. On the entrance of the active site, a peripherical anionic site (PAS) is composed of several aromatic residues (such as Tyr-72, Asp-74, Tyr-124, Trp-286), lining a hydrophobic region that traps ACh and transfers it to the deep catalytic site [41]. Distinctive inhibitors bind to the active center or to the PAS located at the rim of the gorge near the enzyme surface. Several studies have shown that some inhibitors of AChE bind at the catalytic site, acting as competitive inhibitors, while others can bind to PAS located at the rim of the active site gorge. PAS inhibitors inhibit catalysis by sterically blocking ligands from entering and leaving the active site gorge, and by allosterical alteration of the catalytic triad conformation [42]. Two antifouling compounds from *Ascidian Synoicum* described as competitive AChE inhibition with no antibiotic nor cytotoxic activity (pulmonarin A and B) [29] were used as positive controls in the in silico docking study. According to the docking results, pulmonarin A and B are able to bind active site (docking scores: A_(ACTIVE SITE)_ = −5.9 kcal.mol^−1^; B_(ACTIVE SITE)_ = −6.8 kcal.mol^−1^) and PAS (docking scores: A_(PAS)_ = −5.5 kcal.mol^−1^; B_(PAS)_ = −6.8 kcal.mol^−1^). Compound **4** formed a more stable complex (lower docking score) with AChE PAS (docking score: **4**_(PAS)_ = −8.5 kcal.mol^−1^) than positive controls pulmonarin A and B and only formed a more stable complex with AChE active site (docking score: **4**_(ACTIVE SITE)_ = −6.7 kcal.mol^−1^) than pulmonarin A; compound **5** formed a more stable complex (lower docking score) with both AChE PAS and active site (docking score: **5**_(PAS)_ = −7.6 kcal.mol^−1^; **5**_(ACTIVE SITE)_ = −8.2 kcal.mol^−1^) than the positive controls pulmonarin A and B.

In order to understand the binding mode of the most promising molecules **4** and **5** to AChE, a careful examination of the most stable docking poses was performed and compared with controls (Figure 5).

**Figure 6:**
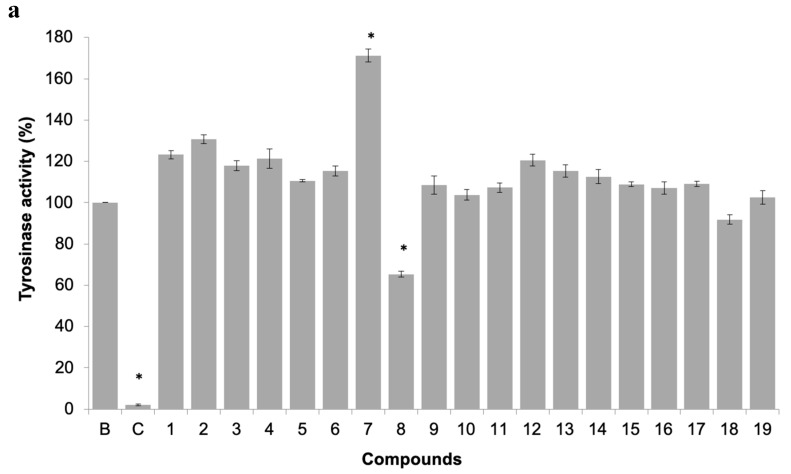

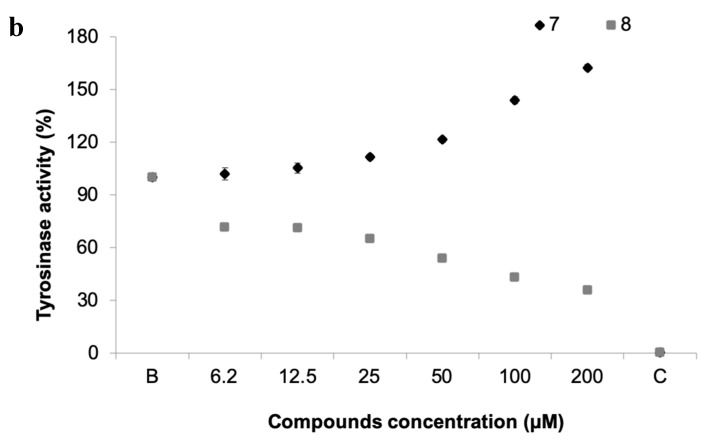
(**a**) Tyrosinase activity of 19 pure xanthones. * significant differences (*p* ≤ 0.05, Dunnett’s test) compared to the negative control (B); kojic acid was used as positive control (C). (**b**) Dose-response tyrosinase activity of selected compounds **7** and **8**.

The interactions between known inhibitors pulmonarin A and B and the enzyme are characterized by hydrogen bonds with residues Asp-74, Gly-120, Gly-121, Gly-122, Tyr-124, Ser-125, Glu-202, Tyr-337, Tyr-341, and His-447 (Figure 6a,b). It is described that ACh binds to Asp-74 at the peripheral site of human AChE as the first step in the catalytic pathway [43]; Gly-120, Gly-121, and Gly-122 are described as important to strengthen the interaction with ACh [44]. Tyr-124 is also shown to be involved in the hydrolysis of ACh, carbamylation, phosphonylation, and oxime reactivation mechanisms, besides regulating the flow of substrates into the catalytic site from the gorge [45]. Ser-125 has already been described as being involved in hydrogen interactions with piperazine AChE inhibitors [46]. The acidic residues Asp-74 and Glu-202 create a dipole that allows the positively charged ACh to move down the gorge [47]. Amino acid residues Tyr-337 and Tyr-341 are involved in the binding of reversible AChE inhibitors Huperzine A [48] and tacrine [49] respectively. His-447 is a member of the catalytic triad, the ring of which is flipped to form a reactive Glu-334-His-447-oxime triad [50].

Compound **5** docked more stably at the bottom of the binding groove that forms a hydrophobic pocket base and that contains the catalytic Ser-203 (docking score of −8.2 kcal.mol^−1^) (Figure 5c), but a higher energy binding pose at PAS also exists (docking score of −7.6 kcal.mol^−1^). On the other hand, compound **4** binds preferably at PAS (Figure 5c), with a docking score of −8.5 kcal.mol^−1^, but binding in the narrow active groove is also possible (docking score of −6.7 kcal.mol^−1^). As far as hydroxyl or methoxy xanthones are concerned, the narrower gorge in AChE results in a conformation where the xanthone scaffold packs against the aromatic hydrophobic portions of the side chain of Trp-86, establishing parallel π-π interactions, as seen for compound **5** (Figure 5c). Packing in this region is quite tight, and only the smallest substituents might be accommodated. Upon analysis of the interaction of compound **5** in AChE active site, hydrogen interactions with Tyr-72, Asp-74, Trp-86, Asn-87, Tyr-124, and Tyr-337 were stablished. The xanthonic moiety of **5** establishes near-parallel stacking of the planar tricyclic ring system with Trp-86 (Figure 5c). As far as compound **4** is concerned, which binds more stably at PAS, it establishes hydrogen interactions with Ser-293 and Tyr-124. The xanthonic moiety of **4** is positioned in a conformation that allows near-parallel stacking of the planar tricyclic ring system with Tyr-341.

All in all, docking results implied that the possible antifouling mechanisms of xanthonic derivatives may be attributed to the interactions between xanthone and AChE, such as hydrogen bonds, π-stacking, cation-dipole, hydrophobic, and van der Waals interactions.

#### 3.3.3. In Vitro Determination of Tyrosinase Activity

The inhibition of this enzyme activity would affect the settlement and attachment ability of mussel larvae, as it has been described as an anti-settlement mode of action [51], however the tyrosinase/phenoxidase cascade pathways play an important role in different physiologically processes in invertebrates, including immune responses, sclerotization of the cuticle and wound healing. From the 19 compounds tested, two compounds were found to modulate tyrosinase activity (Figure 6a). While compound **8** was found to inhibit tyrosinase activity at 34% at 50 µM, the compound **7**, previously discover with anti-settlement activity against mussel larvae, was found to induce tyrosinase activity from 11.7 to 62.5% at 25 to 200 μM, respectively (Figure 6b). Considering this, the induction of tyrosinase activity by compound **7** might be related to accelerated immune responses facing exposure or to higher demand of inputs on the pathways related to the construction of cytoskeleton (adhesive apparatus). This last hypothesis is further supported by the results obtained by differentially expressed proteins in proteomic studies.

#### 3.3.4. Antifouling Targets by Proteomics

**Figure 7:**
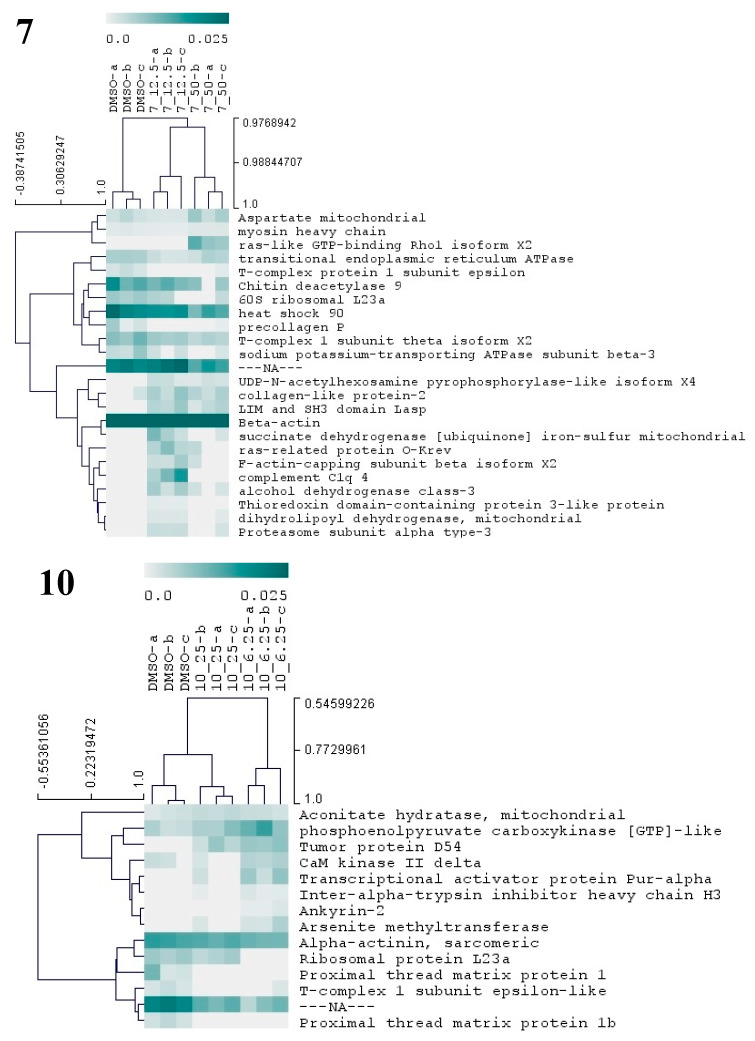

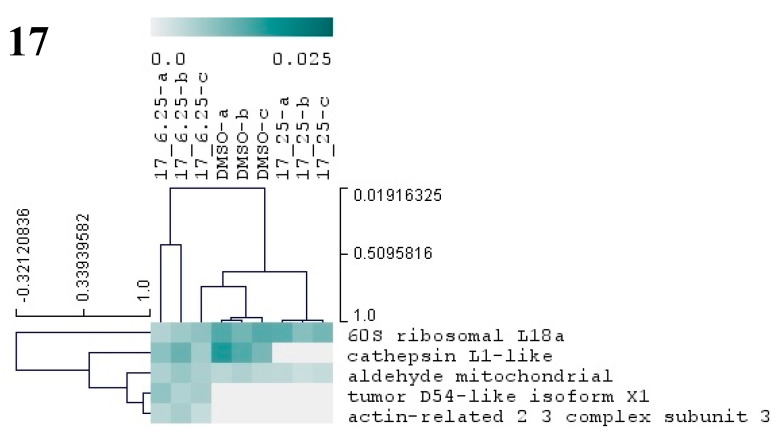
Hierarchical cluster analysis of the differential proteins from *M. galloprovincialis* larvae exposed to three different antifouling compounds, **7**, **10**, and **17**. On the vertical axis of the dendrogram: clustering of proteins with similar abundance profiles. On the horizontal axis: grouping of samples with similar proteome. Only proteins with significant changes (*p* < 0.05) in abundance (based on NSAF values) are presented in the dendogram. The relative abundance values (NSAF) are represented in color gradient from 0.0 to 0.025. Further information on NSAF values, significant p-values, and names of all differentially abundant proteins is included in Supplementary Table S2.

As to provide additional molecular insights on the antifouling properties of selected compounds, the proteome of competent *M. galloprovincialis* plantigrade larvae in response to the three most potent compounds (**7**, **10**, and **17**) was analyzed by label-free shotgun proteomics. Quantitative protein variations were surveyed employing statistics (non-parametric methods, KW and MW) and hierarchical clustering. Independent statistical tests were performed for each individual compound and results are summarized in Figure 7. Supplementary Tables S1 and S2 provide respectively, the complete information regarding the statistics of protein expression and the molecular functions of identified proteins.

Compound **7** was the one that induced more alterations in the mussel plantigrade larvae proteome, among the three antifouling compounds. In total, this compound at 12.5 and 50 µM altered the abundances of 24 proteins (Figure 7, Supplementary Table S1), suggesting alterations in a large spectrum of both general and specialized cellular pathways. The differential proteins are involved in the production of energy (succinate dehydrogenase mitochondrial), cytoskeleton integrity, and vesicle-mediated transport (beta-actin, myosin heavy chain, F-actin-capping subunit beta, LIM and SH3 domain LASP, ras-like GTP-binding Rho1, ras-related protein O-krev, transitional endoplasmic reticulum ATPase), protein folding and chaperone functions (heat shock 90, T-complex 1 subunit theta, T-complex protein 1 subunit epsilon), carbohydrate metabolism (chitin deacetylase 9), amino acid metabolism (aspartate mitochondrial aminotransferase), cell redox homeostasis (alcohol dehydrogenase class-3, thioredoxin domain-containing protein 3, dihydrolipoyl dehydrogenase, mitochondrial), protein glycosylation (UDP-N-acetylhexosamine pyrophosphorylase), gene translation (60S ribosomal L23a), ERK1 and ERK2 mediated signal transduction (complement C1q 4), cellular ion homeostasis and membrane polarization (sodium/potassium-transporting ATPase subunit beta-3) and proteasome activity (proteasome subunit alpha type-3) (Figure 7 and Supplementary Table S2). The results evidence the cellular action of **7** particularly toward the functions of cytoskeleton, chaperone-mediated regulation of protein activity and cell redox status. Two putative collagen proteins, protein-2 collagen-like and precollagen P displayed contrasting patterns of abundance, being respectively above and below the control levels, in the groups exposed to **7**. *Mytilus* collagen proteins (PreCols) are specific to the byssal threads and comprehend the main constituents of this adhesive structure. Several structural domains were identified in PreCols, which are thought to confer increased elasticity in areas of the protein subjected to more tension and bending [52]. Moreover, the byssal thread properties such as resistance to tension and shock absorber are essentially provided by these proteins. The inhibitory effects in adhesion of **7** may well be also associated with the change in the abundance of PreCols.

Exposure of larvae to compound **10** at 6.25 and 25 µM resulted in the change of abundances in 14 proteins (Figure 7, Supplementary Table S1). Taking into consideration the molecular functions attributed to these proteins (Supplementary Figure S2) several cellular responses may be identified, elicited by **10**, which include: alterations in cytoskeleton structure (Alpha-actinin, CaM kinase II, Ankyrin-2), in skeletal muscle function through modulation of Ca^2+^ transport and attachment of integral membrane proteins to cytoskeletal elements (CaM kinase II Ankyrin-2), energy metabolism (phosphoenolpyruvate carboxykinase and aconitate hydratase), gene transcription and translation (Ribosomal protein L23a, transcriptional activator protein Pur-alpha), and regulation of protein activity (tumor protein D54, T-complex 1 epsilon-like subunit). Other altered proteins are hypothetically involved in the transport of hyaluronan (inter-alpha-trypsin inhibitor heavy chain H3), a main component of the extracellular matrix and a key element in cell proliferation and migration, and xenobiotic detoxification processes (arsenite methyltransferase). Two putative proximal thread matrix proteins (TMPs) were clearly detected in the control group but become undetected in the two groups exposed to the compound **10**, herein pointing to a significant drop in the expression of these proteins related to the activity of **10**. TMPs are glycine-, tyrosine-, and asparagine-rich family of proteins carrying unique repeated sequence motifs. The proteins are specifically expressed by bivalve mollusks that adhere to underwater surfaces through the production of byssal threads. TMPs are thought to be localized throughout the byssal threads separating the collagenous microfibrils. Their function is to provide viscoelasticity to the byssal threads [52]. Hence, the inhibition of the two TMPs may be pointed as one of the most critical events underlying inhibition of larvae adhesion by compound **10**.

Exposure of plantigrade larvae to compound **17** at 6.25 and 25 µM altered the abundance of five proteins (Figure 7, Supplementary Table S1). These proteins are known to be involved in major cellular metabolic processes as glycolysis/gluconeogenesis (mitochondrial aldehyde dehydrogenase), gene translation (60S ribosomal protein L18a), lysosomal protein degradation (cathepsin), or are constituents of the cytoskeleton (actin-related 2/3 complex subunit 3) (Figure 7, Supplementary Table S2). A homologous of the tumor protein D54 was detected in larvae exposed to **17** at 6.25 µM. With some of these constitutive proteins playing a role in basic metabolism and cellular functions, it is possible for this compound to affect the metabolism of a broad number of organism.

### 3.4. Non-Target Species Toxicity Assessment

**Figure 8:**
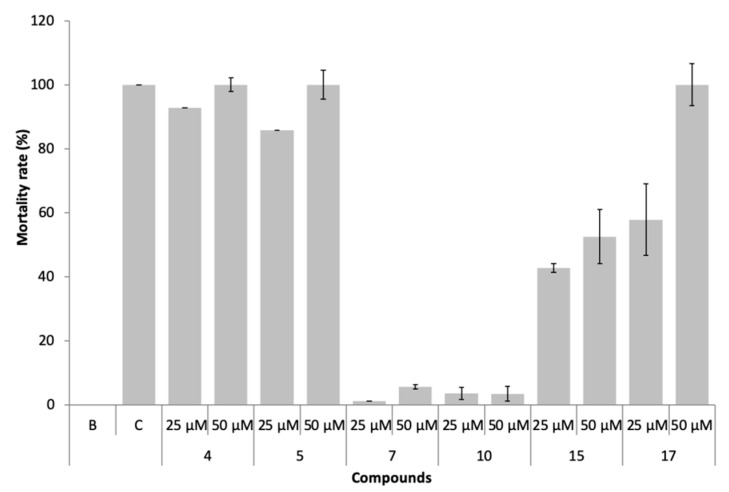
Mortality rate of *Artemia salina* exposed to the compounds **4**, **5**, **7**, **10**, **15**, and **17** at 25 and 50 µM.

The marine ecotoxicity of compounds **4**, **5**, **7**, **10**, **15**, and **17** was studied regarding lethality to the brine shrimp, *Artemia salina* naupliids (Figure 8).

In the presence of compounds **7** and **10**, non-toxic effects (<10% mortality) were observed, even at 50 µM. Compounds **4**, **5, 15**, and **17** were found to be toxic (mortality >10%) to this non-target specie, particularly compounds **4** and **5**.

## 4. Conclusions

The marine industry is facing the phase-out of current persistent, bioaccumulative, and toxic biocides, shortening the available alternatives and creating a great opportunity for the development of new antifouling solutions. One strategy that has been exploited by researchers is the use of chemical defenses naturally employed by several marine organisms, which have been selected during evolution to have high specificity, high efficiency, and also be environmentally compatible inhibitors of biofouling. The synthesis of (bio)inspired antifoulants looks like a more sustainable way bringing an opportunity to produce commercial supplies for antifouling industry based on natural products. In this study, six synthetic xanthone derivatives showed antifouling potential regarding anti-settlement effectiveness and low toxicity to mussel larvae. The IUCN/SSC Invasive Species Specialist Group has listed *M. galloprovincialis* among the 100 “World’s Worst” invaders, highlighting the worldwide relevance of studying the anti-settlement activity against this species. Concerning the mechanisms of action in mussel larvae, compounds **4** and **5** might be acting by the inhibition of AChE activity, while compounds **7** and **10** showed a specific target directly related with the production/constitution of byssal threads (expression of specific collagen proteins (PreCols) and proximal thread proteins (TMPs), respectively). Xanthones **7** and **10** also showed no toxicity (<10% mortality) to *A. salina*, being more suitable candidates to pursue biodegradability and deepen the ecotoxicological studies in the way to their inclusion in antifouling coatings. Also, the QSAR enables speeding the design of new potential active compounds and the synthesis of optimized xanthones with protonable amine groups in other positions is ongoing. In summary, adding to their previously known pharmacological actions, here we disclosed (thio)xanthonic derivatives as efficient antifoulants and brought new insights into their potentially specific mechanism of action related with adhesion.

## Figures and Tables

**Table 1:** Antifouling effectiveness *versus* toxicity of compounds toward the anti-settlement of mussel plantigrades.

Compound	EC_50_ (µM; µg.mL^−1^)	LC_50_ (µM)	LC_50_/EC_50_
**4**	21.48 (95% CI: 15.37–30.79); 4.55	>500	>23.28
**5**	15.46 (95% CI: 10.29–23.30); 3.52	>500	>32.34
**7**	11.53 (95% CI: 7.01–18.39); 2.63	>500	>43.37
**10**	4.60 (95% CI: 3.21–6.20); 1.36	>500	>108.70
**15**	28.60 (95% CI: 19.58–43.97); 12.40	>500	>17.48
**17**	3.53 (95% CI: 1.29–6.47); 1.25	>500	>141.64

EC_50_, minimum concentration that inhibited 50% of larval settlement; LC_50_ the median lethal dose; LC_50_/EC_50,_ therapeutic ratio. Note: reference values for EC_50_ < 25 µg.mL^−1^ (U.S. Navy program) and therapeutic ratio (LC_50_/EC_50_) higher than 15.

## Supplementary Materials

The following are available online at https://www.mdpi.com/2218-273X/10/8/1126/s1, Table S1: NSAF values and Kruskal-wallis p-values of all differentially abundant proteins. Table S2: Blast search results with the tools blast2go and blastp from NCBI.
