# Supplementary material for: Structure-Antifouling Activity Relationship and Molecular Targets of Bio-Inspired(thio)xanthones
Source: Biomolecules. 2020 Jul 30;10(8):1126. doi: 10.3390/biom10081126 (PMC7463931; doi:10.3390/biom10081126)
Supplement: Supplementary file 1 [file biomolecules-10-01126-s001.pdf]

Supplementary material

Structure-antifouling activity relationship and molecular targets of bio-inspired(thio)xanthones

Joana R. Almeida [1], Andreia Palmeira [1,2], Alexandre Campos [1], Isabel Cunha [1], Micaela Freitas[1,3], Aldo Barreiro [1], Maria V Turkina [4], Vitor Vasconcelos [1,3], Madalena Pinto [1,2], Marta Correia-da-Silva[1,2]*, Emília Sousa[1,2]

1 CIIMAR/CIMAR - Interdisciplinary Centre of Marine and Environmental Research, University of Porto, Portugal

2 Laboratory of Organic and Pharmaceutical Chemistry, Department of Chemical Sciences, Faculty of Pharmacy, University of Porto, Rua Jorge Viterbo Ferreira, 228, 4050-313 Porto, Portugal

3 Department of Biology, Faculty of Sciences, University of Porto, Rua do Campo Alegre, P 4069-007 Porto, Portugal

4 Department of Biomedical and Clinical Sciences, Linköping University, SE-581 85, Linköping, Sweden

***Corresponding author:** Laboratory of Organic and Pharmaceutical Chemistry, Department of Chemical Sciences, Faculty of Pharmacy, University of Porto, Rua Jorge Viterbo Ferreira, 228, 4050-313 Porto, Portugal; m_correiadasilva@ff.up.pt

Supplementary Table S1: NSAF values and Kruskal-wallis p-values of all differentially abundant proteins.

		DMSO			**7** _12.5 µM			**7**_50 µM			Kruskal-Wallis	Mann-Whitney (p<0.05)		
sequence reference	protein name	a	b	c	a	b	c	a	b	c	p-value	**7**_12.5µM	**7**_50µM	2_**7**
tr|A8WE66|A8WE66_CRAAR (+1)	Beta-actin	0,108690	0,107580	0,109740	0,130330	0,117350	0,114760	0,107040	0,100730	0,105120	0,027323725			
tr|C0Z203|C0Z203_MYTGA	heat shock 90	0,024013	0,019609	0,018489	0,017300	0,016776	0,017802	0,015008	0,010045	0,013214	0,027323725			
comp77767_c1_seq1	T-complex 1 subunit theta isoform X2	0,009106	0,007436	0,011154	0,007065	0,006362	0,006750	0,005691	0,004444	0,005011	0,027323725			
comp73181_c0_seq1	transitional endoplasmic reticulum ATPase	0,006184	0,006313	0,006060	0,003599	0,002160	0,002292	0,005798	0,003018	0,005105	0,038990222			
comp51760_c0_seq1	---NA---	0,019761	0,021516	0,019366	0,020443	0,023010	0,024416	0,016468	0,012860	0,014499	0,038990222			
comp33963_c0_seq1	Chitin deacetylase 9	0,018145	0,011113	0,013336	0,010559	0,012676	0,010089	0,000000	0,008856	0,007489	0,038990222			
comp75013_c0_seq1	Aspartate mitochondrial	0,002893	0,004726	0,002835	0,002245	0,002021	0,002145	0,003617	0,007532	0,006369	0,038990222			
comp72803_c0_seq1	collagen-like protein-2	0,000000	0,000000	0,002679	0,006363	0,003820	0,008107	0,003417	0,005337	0,006017	0,0496478			
tr|Q26079|Q26079_PLAMG	myosin heavy chain	0,001276	0,001563	0,001251	0,000990	0,000891	0,000946	0,001595	0,001661	0,001756	0,027323725			
comp75613_c0_seq2	F-actin-capping subunit beta isoform X2	0,000000	0,000000	0,000000	0,003520	0,003169	0,006726	0,000000	0,002952	0,000000	0,034735262			
comp66501_c0_seq2	alcohol dehydrogenase class-3	0,000000	0,000000	0,000000	0,006790	0,003057	0,006488	0,000000	0,002848	0,002408	0,033985604			
comp84021_c1_seq3	UDP-N-acetylhexosamine pyrophosphorylase-like isoform X4	0,000000	0,000000	0,000000	0,003703	0,003334	0,001769	0,002983	0,001553	0,002626	0,045938127			
comp83583_c0_seq24	LIM and SH3 domain Lasp	0,000000	0,000000	0,000000	0,005138	0,004627	0,007364	0,004139	0,002155	0,005466	0,045938127			
tr|A0A077H3K4|A0A077H3K4_MYTTR	60S ribosomal L23a	0,007242	0,005914	0,007097	0,005619	0,005060	0,000000	0,000000	0,000000	0,003985	0,045938127			
comp79800_c2_seq1 (+1)	ras-like GTP-binding Rho1 isoform X2	0,000000	0,000000	0,000000	0,000000	0,000000	0,000000	0,008062	0,012592	0,007099	0,022109129			
tr|Q8MW54|Q8MW54_MYTGA	precollagen P	0,006926	0,001131	0,002715	0,000000	0,000000	0,000000	0,000000	0,000000	0,000762	0,034735262			
tr|A0A0B6Z9P2|A0A0B6Z9P2_9EUPU (+2)	ras-related protein O-Krev	0,000000	0,000000	0,000000	0,005222	0,009404	0,004989	0,000000	0,004380	0,000000	0,034735262			
comp76574_c0_seq2	succinate dehydrogenase [ubiquinone] iron-sulfur mitochondrial	0,000000	0,000000	0,000000	0,010222	0,006136	0,003256	0,000000	0,000000	0,002417	0,034735262			
tr|E1UJ66|E1UJ66_MYTGA	complement C1q 4	0,000000	0,000000	0,000000	0,005685	0,010239	0,016297	0,000000	0,000000	0,000000	0,022109129			
comp83424_c0_seq13	Thioredoxin domain-containing protein 3-like protein	0,000000	0,000000	0,000000	0,001182	0,001064	0,001129	0,000000	0,000000	0,000000	0,022109129			
comp76726_c0_seq1	T-complex protein 1 subunit epsilon	0,002289	0,003739	0,002243	0,000000	0,000000	0,000000	0,000000	0,000000	0,001260	0,034735262			
comp77238_c0_seq1	dihydrolipoyl dehydrogenase, mitochondrial	0,000000	0,000000	0,000000	0,001906	0,001717	0,001822	0,000000	0,000000	0,001352	0,034735262			
comp81906_c1_seq4	sodium potassium-transporting ATPase subunit beta-3	0,003995	0,003262	0,007830	0,003100	0,000000	0,002962	0,000000	0,000000	0,002198	0,045938127			
BAGiLS_001001 (+1)	Proteasome subunit alpha type-3	0,000000	0,000000	0,000000	0,003710	0,003340	0,003545	0,000000	0,000000	0,002631	0,034735262			

		DMSO			**10**_6.25			**10**_25			Kruskal-Wallis	Mann-Whitney (p<0.05)		
sequence reference	protein name	a	b	c	a	b	c	a	b	c	p-value	**10**_6.25	**10**_25	2_**10**
comp82344_c0_seq1	Alpha-actinin, sarcomeric	0,015340	0,014805	0,013666	0,011490	0,010775	0,010632	0,011820	0,013203	0,013287	0,027323725			
comp83803_c0_seq1	phosphoenolpyruvate carboxykinase [GTP]-like	0,005823	0,003170	0,003804	0,011631	0,014997	0,008221	0,005982	0,006126	0,009247	0,038990222			
comp80593_c1_seq1	Aconitate hydratase, mitochondrial	0,001584	0,002586	0,003104	0,003559	0,003337	0,002683	0,003660	0,003998	0,004527	0,038990222			
comp51760_c0_seq1	---NA---	0,019761	0,021516	0,019366	0,004934	0,009254	0,011160	0,010151	0,012473	0,012552	0,038990222			
comp82829_c0_seq11	Tumor protein D54	0,000000	0,000000	0,000000	0,007603	0,007130	0,008598	0,007821	0,003203	0,004836	0,045938127			
tr|A0A077H3K4|A0A077H3K4_MYTTR	Ribosomal protein L23a	0,007242	0,005914	0,007097	0,000000	0,000000	0,000000	0,005580	0,004571	0,006900	0,034863256			
tr|Q8T5C2|Q8T5C2_MYTGA	Proximal thread matrix protein 1	0,010935	0,002232	0,002679	0,000000	0,000000	0,000000	0,000000	0,000000	0,000000	0,022109129			
comp77028_c0_seq2	CaM kinase II delta	0,003548	0,002898	0,000000	0,005316	0,004985	0,006012	0,000000	0,002240	0,000000	0,045938127			
comp76726_c0_seq1	T-complex 1 subunit epsilon-like	0,002289	0,003739	0,002243	0,000000	0,000000	0,001939	0,000000	0,000000	0,000000	0,034735262			
comp84186_c0_seq15	Transcriptional activator protein Pur-alpha	0,000000	0,000000	0,000000	0,007333	0,003438	0,008293	0,000000	0,003090	0,000000	0,034735262			
comp84151_c0_seq10	Ankyrin-2	0,000000	0,000000	0,000000	0,000853	0,000800	0,001930	0,000000	0,000000	0,000000	0,022109129			
comp77154_c0_seq1	Inter-alpha-trypsin inhibitor heavy chain H3	0,000000	0,000000	0,000000	0,002096	0,000983	0,001185	0,000000	0,000883	0,000000	0,034735262			
comp81375_c0_seq1	Arsenite methyltransferase	0,000000	0,000000	0,000000	0,002548	0,002390	0,005764	0,000000	0,002147	0,000000	0,034735262			
tr|Q8T5C3|Q8T5C3_MYTED	Proximal thread matrix protein 1b	0,002789	0,004555	0,002733	0,000000	0,000000	0,000000	0,000000	0,000000	0,000000	0,022109129			

		dmso			**17**_6.25			**17**_25			Kruskal-Wallis	Mann-Whitney (p<0.05)		
sequence reference	protein name	a	b	c	a	b	c	a	b	c	p-value	**17**_6.25	**17**_25	2_**17**
comp71715_c0_seq1	aldehyde mitochondrial	0,004763	0,005834	0,004668	0,005852	0,007738	0,006137	0,004568	0,003317	0,003837	0,027323725			
comp64527_c0_seq1	60S ribosomal L18a	0,013174	0,010758	0,012910	0,005396	0,007134	0,008487	0,012634	0,009176	0,010612	0,038990222			
comp82829_c0_seq11	tumor D54-like isoform X1	0,000000	0,000000	0,000000	0,008315	0,005497	0,006539	0,000000	0,000000	0,000000	0,022109129			
comp62389_c0_seq2	cathepsin L1-like	0,015876	0,012965	0,010372	0,008670	0,011464	0,006819	0,000000	0,000000	0,000000	0,034863256			
BAGiLS_002246 (+1)	actin-related 2 3 complex subunit 3	0,000000	0,000000	0,000000	0,004831	0,006387	0,003799	0,000000	0,000000	0,000000	0,022109129			

Supplementary Table S2: Blast search results with the tools blast2go and blastp from NCBI

Compound 7 differential proteins

sequence reference	eValue	accession	annotation	species	functions
tr|A8WE66|A8WE66_CRAAR (+1)	0,00E+00	A8WE66	Beta-actin	Crassostrea ariakensis	F:ATP binding; C:cytoskeleton; C:cytoplasm
tr|C0Z203|C0Z203_MYTGA	0,00E+00	C0Z203_MYTGA	heat shock 90	Mytilus galloprovincialis	F:ATP binding; F:unfolded protein binding; P:protein folding; P:response to stress
comp77767_c1_seq1	0,00E+00	gi|405961548	T-complex 1 subunit theta isoform X2	Crassostrea gigas	F:ATP binding; C:microtubule organizing center; C:cytoplasm; F:unfolded protein binding; P:protein folding
comp73181_c0_seq1	0,00E+00	gi|762083723	transitional endoplasmic reticulum ATPase	Crassostrea gigas	P:chordate embryonic development; C:site of double-strand break; F:lipid binding; P:transport; P:cell cycle; P:double-strand break repair; C:nucleus; F:ATP binding; P:locomotory behavior; P:regulation of protein ubiquitination involved in ubiquitin-dependent protein catabolic process; C:cytosol; P:protein N-linked glycosylation via asparagine; P:ER-associated ubiquitin-dependent protein catabolic process; P:translesion synthesis; F:hydrolase activity; P:protein ubiquitination
comp51760_c0_seq1	-		---NA---		
comp33963_c0_seq1	1,50E-33	A0A0P5IKY1	Chitin deacetylase 9	Daphnia magna	F:chitinase activity; F:hydrolase activity, acting on carbon-nitrogen (but not peptide) bonds; P:carbohydrate metabolic process; F:chitin binding; P:chitin catabolic process; C:extracellular region
comp75013_c0_seq1	0,00E+00	gi|405973112	Aspartate aminotransferase mitochondrial	Crassostrea gigas	P:aspartate biosynthetic process; F:poly(A) RNA binding; P:aspartate catabolic process; P:response to ethanol; P:oxaloacetate metabolic process; C:extracellular exosome; P:fatty acid transport; P:glutamate catabolic process to 2-oxoglutarate; P:glutamate catabolic process to aspartate
comp72803_c0_seq1	0,00E+00	gi|906541694	collagen-like protein-2	Mytilus coruscus	F:zinc ion binding; C:collagen trimer; P:metabolic process; F:ligase activity
tr|Q26079|Q26079_PLAMG	0,00E+00	Q26079_PLAMG	myosin heavy chain	Placopecten magellanicus	F:ATP binding; F:calmodulin binding; F:actin binding; P:metabolic process; C:myosin filament; C:myofibril; F:motor activity
comp75613_c0_seq2	5,90E-169	gi|41053959	F-actin-capping subunit beta isoform X2	Danio rerio	F:actin filament binding; P:regulation of lamellipodium assembly; P:barbed-end actin filament capping; C:cytoplasm; P:cell morphogenesis; P:negative regulation of filopodium assembly; C:actin filament; C:F-actin capping protein complex; P:actin cytoskeleton organization
comp66501_c0_seq2	1,68E-116	gi|405972460	alcohol dehydrogenase class-3	Crassostrea gigas	F:zinc ion binding; F:S-(hydroxymethyl)glutathione dehydrogenase activity; P:ethanol oxidation
comp84021_c1_seq3	0,00E+00	gi|762098431	UDP-N-acetylhexosamine pyrophosphorylase-like isoform X4	Crassostrea gigas	F:identical protein binding; C:cytoplasm; P:metabolic process; C:plasma membrane; C:nucleoplasm; F:uridylyltransferase activity
comp83583_c0_seq24	2,56E-75	gi|345491291	LIM and SH3 domain Lasp	Nasonia vitripennis	F:zinc ion binding
tr|A0A077H3K4|A0A077H3K4_MYTTR	0,00E+00	A0A077H3K4_MYTTR	60S ribosomal L23a	Mytilus trossulus	F:nucleotide binding; F:structural constituent of ribosome; C:ribosome; P:translation
comp79800_c2_seq1 (+1)	2,20E-135	gi|333449487	ras-like GTP-binding Rho1 isoform X2	Crassostrea ariakensis	F:GTP binding; P:nucleocytoplasmic transport; P:small GTPase mediated signal transduction; F:GTPase activity; P:metabolic process; P:intracellular protein transport; C:intracellular; C:plasma membrane
tr|Q8MW54|Q8MW54_MYTGA	0,00E+00	Q8MW54_MYTGA	precollagen P	Mytilus galloprovincialis	C:collagen trimer
tr|A0A0B6Z9P2|A0A0B6Z9P2_9EUPU (+2)	7,10E-105	gi|432866740	ras-related protein O-Krev	Oryzias latipes	F:GTP binding; P:nucleocytoplasmic transport; P:small GTPase mediated signal transduction; F:GTPase activity; C:membrane; P:metabolic process; P:intracellular protein transport; C:intracellular
comp76574_c0_seq2	1,05E-159	gi|961134210	succinate dehydrogenase [ubiquinone] iron-sulfur mitochondrial	Octopus bimaculoides	F:succinate dehydrogenase (ubiquinone) activity; F:metal ion binding; F:electron carrier activity; F:3 iron, 4 sulfur cluster binding; P:tricarboxylic acid cycle; F:4 iron, 4 sulfur cluster binding; F:2 iron, 2 sulfur cluster binding; C:mitochondrial inner membr
tr|E1UJ66|E1UJ66_MYTGA	2,60E-122	gi|405957207	complement C1q 4	Crassostrea gigas	F:oxidoreductase activity, acting on CH-OH group of donors; F:flavin adenine dinucleotide binding; C:integral component of membrane; F:protein dimerization activity; P:oxidation-reduction process
comp83424_c0_seq13	0,00E+00	gi|405969114	Thioredoxin domain-containing protein 3-like protein	Crassostrea gigas	C:cell; P:UTP biosynthetic process; P:CTP biosynthetic process; P:GTP biosynthetic process; P:nucleoside diphosphate phosphorylation; P:cell adhesion; P:cell redox homeostasis; F:nucleoside diphosphate kinase activity; F:carbohydrate binding
comp76726_c0_seq1	0,00E+00	gi|405970171	T-complex protein 1 subunit epsilon	Crassostrea gigas	F:ATP binding; C:cytoplasm; F:unfolded protein binding; P:protein folding
comp77238_c0_seq1	0,00E+00	gi|723570344	dihydrolipoyl dehydrogenase, mitochondrial	Balearica regulorum gibbericeps	C:cell; F:flavin adenine dinucleotide binding; P:cell redox homeostasis; P:oxidation-reduction process; F:dihydrolipoyl dehydrogenase activity
comp81906_c1_seq4	1,20E-91	gi|762104468	sodium potassium-transporting ATPase subunit beta-3	Crassostrea gigas	P:potassium ion transport; P:sodium ion transport; C:sodium:potassium-exchanging ATPase complex
BAGiLS_001001 (+1)	8,30E-151	gi|762126183	Proteasome subunit alpha type-3	Crassostrea gigas	C:nucleus; C:cytoplasm; C:proteasome core complex, alpha-subunit complex; F:threonine-type endopeptidase activity; P:ubiquitin-dependent protein catabolic process

Compound 10 differential proteins

sequence reference	eValue	accession	annotation	species	functions
comp82344_c0_seq1	0,00E+00	gi|405978716	Alpha-actinin, sarcomeric	Crassostrea gigas	F:actin filament binding; P:actin crosslink formation; F:calcium ion binding; P:actin filament bundle assembly
comp83803_c0_seq1	0,00E+00	gi|762070884	phosphoenolpyruvate carboxykinase [GTP]-like	Crassostrea gigas	F:GTP binding; P:gluconeogenesis; F:phosphoenolpyruvate carboxykinase (GTP) activity; P:phosphorylation; F:kinase activity
comp80593_c1_seq1	0,00E+00	gi|762118955	Aconitate hydratase, mitochondrial	Crassostrea gigas	F:metal ion binding; C:mitochondrion; P:tricarboxylic acid cycle; F:4 iron, 4 sulfur cluster binding; F:aconitate hydratase activity
comp51760_c0_seq1			---NA---		
comp82829_c0_seq11	8,30E-79	K1R5P2	Tumor protein D54	Crassostrea gigas	regulation of cell proliferation, protein binding, protein homodimerization activity, poly(A) RNA binding, protein heterodimerization activity, perinuclear region of cytoplasm
tr|A0A077H3K4|A0A077H3K4_MYTTR	0,00E+00	A0A077H3K4_MYTTR	Ribosomal protein L23a	Mytilus trossulus	F:nucleotide binding; F:structural constituent of ribosome; C:ribosome; P:translation
tr|Q8T5C2|Q8T5C2_MYTGA	0,00E+00	Q8T5C2_MYTGA	Proximal thread matrix protein 1	Mytilus galloprovincialis	F:metal ion binding; proteinaceous extracellular matrix
comp77028_c0_seq2	0,00E+00	gi|325297015	CaM kinase II delta	Aplysia californica	F:ATP binding; F:calmodulin binding; F:calmodulin-dependent protein kinase activity; P:protein phosphorylation
comp76726_c0_seq1	0,00E+00	gi|405970171	T-complex 1 subunit epsilon-like	Crassostrea gigas	F:ATP binding; C:cytoplasm; F:unfolded protein binding; P:protein folding
comp84186_c0_seq15	1,40E-150	K1RWS2	Transcriptional activator protein Pur-alpha	Crassostrea gigas	Activator, Transcription, Transcription regulation, DNA-binding
comp84151_c0_seq10	0,00E+00	K1Q086_CRAGI	Ankyrin-2	Crassostrea gigas	membrane proteins to cytoskeletal elements. Also binds to cytoskeletal proteins. Required for coordinate assembly of Na/Ca exchanger, Na/K ATPase and InsP3 receptor at sarcoplasmic reticulum sites in cardiomyocytes. Required for the coordinated expression of the Na/K ATPase, Na/Ca exchanger and beta-2-spectrin (SPTBN1) in the inner segment of rod photoreceptors. Required for expression and targeting of SPTBN1 in neonatal cardiomyocytes and for the regulation of neonatal cardiomyocyte
comp77154_c0_seq1	0,00E+00	K1PXQ1	Inter-alpha-trypsin inhibitor heavy chain H3	Crassostrea gigas	serine-type endopeptidase inhibitor activity, hyaluronan metabolic process
comp81375_c0_seq1	3,80E-134	E3TE24 (E3TE24_ICTPU)	Arsenite methyltransferase	*Ictalurus punctatus*	methyltransferase activity, arsenite methyltransferase activity, cytosol
tr|Q8T5C3|Q8T5C3_MYTED	0,00E+00	Q8T5C3_MYTED	Proximal thread matrix protein 1b	Mytilus edulis	F:metal ion binding; proteinaceous extracellular matrix

Compound 17 differential proteins

sequence reference	eValue	accession	annotation	species	functions
comp71715_c0_seq1	0,00E+00	gi|762077372	aldehyde dehydrogenase mitochondrial	Crassostrea gigas	F:aldehyde dehydrogenase (NAD) activity; P:carbohydrate metabolic process; C:extracellular exosome; C:mitochondrial matrix; F:aldehyde dehydrogenase [NAD(P)+] activity; F:electron carrier activity; P:ethanol catabolic process; P:ethanol oxidation
comp64527_c0_seq1	1,50E-102	gi|762084638	60S ribosomal protein L18a	Crassostrea gigas	F:structural constituent of ribosome; C:membrane; C:cytosolic large ribosomal subunit; F:poly(A) RNA binding; P:translation
comp82829_c0_seq11	8,30E-79	K1R5P2	Tumor protein D54	Crassostrea gigas	regulation of cell proliferation, protein binding, protein homodimerization activity, poly(A) RNA binding, protein heterodimerization activity, perinuclear region of cytoplasm
comp62389_c0_seq2	5,33E-98	gi|762096657	cathepsin L1-like	Crassostrea gigas	P:proteolysis; F:cysteine-type peptidase activity
BAGiLS_002246	4,40E-100	gi|765826393	actin-related 2 3 complex subunit 3	Crassostrea gigas	F:actin filament binding; C:extracellular exosome; C:focal adhesion; C:cytoplasm; C:membrane; P:Arp2/3 complex-mediated actin nucleation; F:structural constituent of cytoskeleton; C:Arp2/3 protein complex; C:lamellipodium
